# A Cross-Sectional Study of Catheter-Related Bloodstream Infections in a Tertiary Care Hospital in India

**DOI:** 10.7759/cureus.81868

**Published:** 2025-04-08

**Authors:** Nirmala Poddar, Roshni Dandapat, Jyoti Prakash Sahoo, Sujit Pradhan, Adrita Das, Alpana Mishra, Dipti Pattnaik

**Affiliations:** 1 Microbiology, Kalinga Institute of Medical Sciences, Bhubaneswar, IND; 2 Pharmacology, Kalinga Institute of Medical Sciences, Bhubaneswar, IND; 3 Infectious Diseases, Kalinga Institute of Medical Sciences, Bhubaneswar, IND; 4 Community Medicine, Kalinga Institute of Medical Sciences, Bhubaneswar, IND

**Keywords:** alluvial plot, antibiotics therapy, antimicrobial susceptibility test, bloodstream infection, catheter colonization, catheter contamination, catheter-related blood stream infection, central line-associated bloodstream infection, correlation analysis, mosaic plot

## Abstract

Background and objectives: Catheter-related bloodstream infections (CRBSI) are commonly seen in critically ill patients with indwelling central venous catheters. To address CRBSI, one must know the causative microorganisms and their antimicrobial susceptibility profiles. This study aimed to identify the microbes that cause CRBSI and their antimicrobial susceptibility patterns.

Methods: This cross-sectional study was conducted at the Kalinga Institute of Medical Sciences (KIMS), Bhubaneswar, India, between March 2022 and February 2023. Adult patients in the ICU with a central line and suspected CRBSI during the study period were included. Those with a bloodstream infection (BSI) prior to ICU admission were excluded. Two blood samples from peripheral veins and the lumen of a central venous catheter were collected for culture and sensitivity of the microorganisms. Chocolate agar, 5% sheep blood agar, and MacConkey agar served to culture pathogenic microbes. The VITEK 2 system (bioMérieux, Marcy-l'Étoile, France) was used to determine the pathogenic strains and perform antimicrobial susceptibility testing (AST). We used the R software (R Foundation for Statistical Computing, Vienna, Austria, version 4.4.3) to analyze the data.

Results: Among the 84 participants, CRBSI was found in 36 (42.8%) patients. Their median age was 53.5 (45.8-65.0) years. Of those 36 patients, 25 (69.4%) were males. The most common cannulation site was the femoral vein (14, 38.9%), followed by the internal jugular vein (12, 33.3%) and the subclavian vein (10, 27.8%). The median durations of ICU stay and indwelling catheters were 23.0 (13.8-41.3) days and 16.0 (8.8-30.0) days, respectively. The most common microorganism causing CRBSI was *Klebsiella pneumoniae* (8, 22.2%), followed by *Acinetobacter baumannii* (5, 13.9%), *Burkholderia cepacia* (3, 8.3%), *Candida tropicalis* (3, 8.3%), *Escherichia coli *(3, 8.3%), *Pseudomonas aeruginosa *(3, 8.3%), and *Staphylococcus aureus *(3, 8.3%). *Klebsiella pneumoniae* and *Escherichia coli *specimens were highly sensitive to tigecycline. *Acinetobacter baumannii*, *Burkholderia cepacia*, and *Pseudomonas aeruginosa *specimens were mainly sensitive to colistin, tigecycline, and ertapenem. *Staphylococcus aureus* isolates were sensitive to vancomycin, linezolid, and daptomycin. *Candida tropicalis* isolates were 100% sensitive to caspofungin and micafungin.

Conclusion: CRBSI among our study participants was mainly caused by *Klebsiella pneumoniae*, *Acinetobacter baumannii*, *Burkholderia cepacia*, *Escherichia coli*, *Pseudomonas aeruginosa*, *Staphylococcus aureus*, and* Candida tropicalis*. The Gram-negative bacteria were highly susceptible to tigecycline. *Staphylococcus aureus* specimens demonstrated their sensitivity to vancomycin, linezolid, and daptomycin. The *Candida tropicalis* specimens were sensitive to echinocandins. We suggest further studies with more participants to investigate the pathogens causing CRBSI and their AST patterns.

## Introduction

Patients with critical conditions in the intensive care units (ICUs) receive treatment frequently through central venous catheters. Large veins such as the femoral vein (FV), subclavian vein (SCV), and internal jugular vein (IJV) are typically utilized for routing these central lines [1-3]. The distal segment of the central line eases access to the inferior or superior vena cava. Although venous access is simple, bloodstream infections (BSI) are frequently triggered by these venous catheters [1,4,5]. Morbidity, mortality, and prolonged hospital stay are all linked to catheter-related bloodstream infections (CRBSI) [1,2,6,7]. However, the incidence of CRBSI decreased after hand hygiene, aseptic and antiseptic skin preparation, and catheter site care were implemented [7-9].

Systemic infection occurring in patients with indwelling venous catheters is regarded as BSI. Bacteremia with the same microorganism detected in the peripheral vein and central line lumen on the same day is regarded as CRBSI [6,10,11]. The common clinical features of CRBSI are spiking fever, hypotension, unexplained tachycardia, local inflammation, purulence, and thrombophlebitis of central veins [11]. The use of corticosteroids and neutropenia might reduce these signs and symptoms. CRBSI in neutropenic patients can be seen without swelling or purulence at the insertion site [10-13]. Asymptomatic patients with a positive catheter-site culture and a negative blood culture from any peripheral vein are referred to as colonizers [11].

The most common pathogens involved in BSI or CRBSI are *Klebsiella pneumoniae*, *Acinetobacter baumannii*, *Burkholderia cepacia*, *Escherichia coli*, *Pseudomonas aeruginosa*, and *Staphylococcus aureus* [11,13-15]. Two recent studies found *Serratia marcescens* and *Candida albicans* in patients with CRBSI [16,17]. An effective preventative strategy can be recommended if the pertinent CRBSI determinants and the appropriate antibiogram of the causing pathogens are known. Hence, we planned this study to determine the microbiological profile of the patients with CRBSI and their antimicrobial susceptibility patterns. We also evaluated the association between the duration of the ICU stay and the central line.

## Materials and methods

Patients with suspected CRBSI had their culture reports scrutinized for pathogens and antimicrobial susceptibility testing (AST) patterns. Between March 2022 and February 2023, the study was conducted at the Kalinga Institute of Medical Sciences (KIMS) in Bhubaneswar, India. On January 13, 2022, we got ethical clearance from our Institutional Ethics Committee (KIIT/KIMS/IEC/794/2022). The study complied with good laboratory practices, the Declaration of Helsinki, and institutional norms.

Study participants

Adult patients admitted to the medicine ICU during the designated time frame, had a central venous catheter inserted, and experienced bacteremia following their ICU stay were included in this cross-sectional study. Patients under the age of 18, those who had BSI prior to ICU admission, any obvious source of systemic infection, and those who were not given a central line throughout their ICU stay were all excluded.

Case definitions

Catheter-Related Bloodstream Infections

The participants who had a fever at least 48 hours after a central line was inserted and exhibited discharge, swelling, or redness at the insertion site were deemed to have suspected CRBSI. A differential time to positivity (DTP) of more than two hours was used to diagnose CRBSI when the same organism was cultured from the blood culture of the peripheral veins and the central line. CRBSI implies a positive central line culture is acquired at least two hours before a peripheral vein culture [6,18,19].

Colonizer

The participants with a sterile peripheral line and a microorganism growing in the central line were considered colonizers [6,11].

Bloodstream Infections

The participants with positive culture reports of the same pathogen from both central line and peripheral veins with a DTP shorter than two hours were considered to have BSI. The definition also covered the participants' growth of different microorganisms in the central and peripheral veins [6,10,11].

Procedure

The demographic (e.g., gender, age) and clinical parameters (e.g., central line insertion site, durations of ICU stay and indwelling catheter, purpose for central line insertion, development of septic shock, pathogen, and outcome) of all participants were noted. We grouped the study participants as per the case definitions (i.e., CRBSI, colonizer, and BSI), central line insertion sites, age group (young: < 60 years, elderly: ≥ 60 years), and gender (female and male).

Blood culture

Under aseptic and antiseptic measures, two 10 ml samples for blood culture were collected from the peripheral vein and central lines, as the participants were suspected to have CRBSI. These blood culture bottles were placed within the BACTEC (Becton, Dickinson and Company (BD), New Jersey, USA) apparatus and allowed to incubate. The bottles that were marked positive by the BACTEC were analyzed for Gram staining. Then they were cultured on appropriate media, such as MacConkey agar, chocolate agar, and 5% sheep blood agar.

VITEK 2 system

Initial identification was done using enzymatic tests such as oxidase, coagulase, and catalase. Using the Clinical and Laboratory Standard Institute (CLSI) 2022 cut-off values [20], the VITEK 2 system (bioMérieux, Marcy-l'Étoile, France) was used to identify isolates and evaluate antibiotic susceptibility. The colorimetric reagent card used in the VITEK 2 system had 64 wells. Yeast identification (YST), Gram-negative identification (GN), and Gram-positive identification (GP) cards were used to identify yeast, Gram-negative, and Gram-positive bacteria. The microorganisms in the well were identified by their metabolic activities and by comparing their response patterns to a database.

Antimicrobial susceptibility testing

It works by dilution of micro-broth. In the card wells, the antimicrobial agents were diluted twice. The well was injected with the microorganism suspension. The highest dilution at which an antimicrobial inhibits the microorganism's growth is the minimum inhibitory concentration (MIC). After 18 to 24 hours, colonies were removed from the agar plates and processed using saline solution or broth. The suspension turbidity was controlled to 0.5 McFarland using a calibrated photometric device (Densichek, bioMérieux, Marcy-l'Étoile, France). A filling tube automatically injected the AST inoculum with a predetermined antimicrobial concentration into the 64-well colorimetric reagent card. Optical readings were taken every 15 minutes while the cards were incubated to evaluate light transmission, including the growth control well, across the wells. Each well's MIC data and growth kinetics were interpreted using validated software. The Advanced Expert System (AES) confirmed the final MIC result and antimicrobial susceptibility.

Statistical analysis

This cross-sectional study was conducted using convenience sampling. The data distribution's normality was gauged using the Shapiro-Wilk test. The median and interquartile range (IQR) interpreted the continuous data. Frequency and proportion expressed the categorical data. We used a mosaic plot to illustrate the distribution of the study population. Through an alluvial plot, we portrayed the pathogens and fate of the participants. We used Pearson's correlation to determine the association between the duration of ICU stay and the indwelling central line. For the data analysis, R software (R Foundation for Statistical Computing, Vienna, Austria) version 4.4.3 was used [21]. Statistical significance was set at the p-value of 0.05 or less.

## Results

A total of 1183 patients were admitted to the medicine ICU of our hospital during the study period. Of them, one hundred fifty-seven subjects were below 18 years of age. Two hundred sixty-one individuals had developed BSI before their ICU admission. Three hundred sixty-eight did not necessitate central line insertion during their ICU stay. Two hundred eighty-six patients died within 48 hours of their ICU admission. Twenty-seven patients did not develop any symptoms of CRBSI and had sterile central lines. The remaining 84 patients had a fever and purulent discharge from the insertion site of the central line after 48 hours of cannulation. They were suspected to have CRBSI, and their data were analyzed in this cross-sectional study. Table 1 shows the sociodemographic and clinical traits of the study participants. The median age of the study population was 59.5 (49.5-67.3) years. Fifty-six (66.7%) participants were males. Among the 84 participants, CRBSI prevailed (36, 42.8%), followed by colonizer (30, 35.6%) and BSI (18, 21.6%). The most common cannulation site for the central line was the internal jugular vein (34, 40.5%), followed by the femoral vein (28, 33.3%) and the subclavian vein (22, 26.2%). The median duration of the in situ central line was 15.5 (9.0-30.0) days. The median duration of ICU stay was 25.0 (14.8-40.0) days. The most common pathogens noticed in our study population were *Klebsiella pneumoniae* (19, 22.6%), *Acinetobacter baumannii* (14, 16.7%), *Burkholderia cepacia* (12, 14.3%), *Escherichia coli* (10, 11.9%), *Pseudomonas aeruginosa* (6, 7.1%), *Enterobacter cloacae complex* (6, 7.1%), and *Staphylococcus aureus* (6, 7.1%). Most parameters were similar across the three groups except for age, pathogen, and durations of in situ cannula and ICU stay.

**Table 1:** Sociodemographic and clinical parameters of the study population The continuous variables were expressed as the median and interquartile range. The categorical variables were expressed as frequency and proportion. CRBSI: catheter-related bloodstream infection; BSI: bloodstream infection; FV: femoral vein; IJV: internal jugular vein; SCV: subclavian vein; ICU: intensive care unit; TPN: total parenteral nutrition Statistical significance was set at a p-value of 0.05 or less. p ≥ 0.05 indicates a non-significant difference, the ‘*’ sign indicates a significant difference (p-value < 0.05), the ‘**’ sign indicates a very significant difference (p-value < 0.01), and the ‘***’ sign indicates a highly significant difference (p-value < 0.001).

Parameter	Total (n = 84)	CRBSI (n = 36)	Colonizer (n = 30)	BSI (n = 18)	Test statistics	p-value
Age (in years)	59.5 (49.5 – 67.3)	53.5 (45.8 – 65.0)	60.0 (47.0 – 67.0)	67.5 (52.0 – 71.8)	359.47	< 0.001***
Male	56 (66.7%)	25 (69.4%)	20 (66.7%)	11 (61.1%)	12.82	0.98
Elderly (age > 60 years)	38 (45.2%)	13 (36.1%)	14 (46.7%)	11 (61.1%)	14.33	0.96
Cannulation site						
FV	28 (33.3%)	14 (38.9%)	9 (30.0%)	5 (27.8%)	25.70	0.94
IJV	34 (40.5%)	12 (33.3%)	13 (43.3%)	9 (50.0%)		
SCV	22 (26.2%)	10 (27.8%)	8 (26.7%)	4 (22.2%)		
Duration of in situ venous catheter	15.5 (9.0 – 30.0)	16.0 (8.8 – 30.0)	14.0 (8.3 – 30.0)	18.0 (9.3 – 27.0)	773.99	< 0.001***
Duration of ICU stay	25.0 (14.8 – 40.0)	23.0 (13.8 – 41.3)	23.0 (14.5 – 38.5)	26.5 (20.0 – 38.8)	745.76	< 0.001***
Central line insertion for volume resuscitation	39 (46.4%)	14 (38.9%)	13 (43.3%)	12 (66.7%)	33.61	0.58
Central line insertion for TPN	51 (60.7%)	24 (66.7%)	18 (60.0%)	9 (50.0%)	24.39	0.83
Septic shock	21 (25.0%)	11 (30.6%)	7 (23.3%)	3 (16.7%)	18.42	0.91
Pathogen						
Acinetobacter baumannii	14 (16.7%)	5 (13.9%)	6 (20.0%)	3 (16.7%)	175.16	< 0.001***
Burkholderia cepacia	12 (14.3%)	3 (8.3%)	6 (20.0%)	3 (16.7%)		
Candida albicans	2 (2.4%)	2 (5.6%)	0	0		
Candida tropicalis	5 (6.0%)	3 (8.3%)	1 (3.3%)	1 (5.6%)		
*Enterobacter cloacae *complex	6 (7.1%)	2 (5.6%)	2 (6.7%)	2 (11.1%)		
Enterococcus faecium	2 (2.4%)	2 (5.6%)	0	0		
Escherichia coli	10 (11.9%)	3 (8.3%)	7 (23.3%)	0		
Klebsiella pneumoniae	19 (22.6%)	8 (22.2%)	5 (16.7%)	6 (33.3%)		
Providencia stuartii	1 (1.2%)	1 (2.7%)	0	0		
Pseudomonas aeruginosa	6 (7.1%)	3 (8.3%)	3 (10.0%)	0		
Serratia marcescens	1 (1.2%)	1 (2.7%)	0	0		
Staphylococcus aureus	6 (7.1%)	3 (8.3%)	0	3 (16.7%)		
Outcome						
Discharge	53 (63.1%)	21 (58.3%)	19 (63.3%)	13 (72.2%)	36.29	0.52
Death	31 (36.9%)	15 (41.7%)	11 (36.7%)	5 (28.8%)		

**Figure 1:**
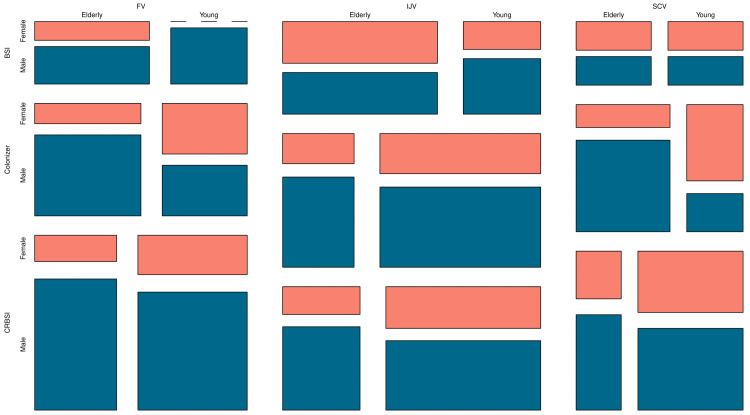
Distribution of the study population The mosaic plot illustrates the distribution of the study population as per their case definition (i.e., CRBSI, colonizer, or BSI), site of cannulation (FV, IJV, or SCV), gender (male or female), and age group (elderly and young individuals). CRBSI: catheter-related bloodstream infection; BSI: bloodstream infection; FV: femoral vein; IJV: internal jugular vein; SCV: subclavian vein

The mosaic plot in Figure 1 illustrates the distribution of the study participants. The parameters employed for grouping were cannulation site (FV, IJV, or SCV), case definition (CRBSI, colonizer, or BSI), age group (elderly and young individuals), and gender (male or female). First, the plot was divided according to the cannulation site. Subsequently, the participants were grouped as per their diagnosis, age group, and gender. It was obvious from the plot that there was a male preponderance in the study population. The most common cannulation site was IJV, followed by FV and SCV. Six young males with central lines in FV had CRBSI. Similarly, six young males with central lines in the IJV were colonizers. Five elderly males with central lines in FV had CRBSI. Five young males with central lines in the IJV had CRBSI. Four young males with central lines in SCV had CRBSI. Four elderly females with central lines in FV and SCV were colonizers. No young female patient was cannulated through FV and had BSI.

**Figure 2:**
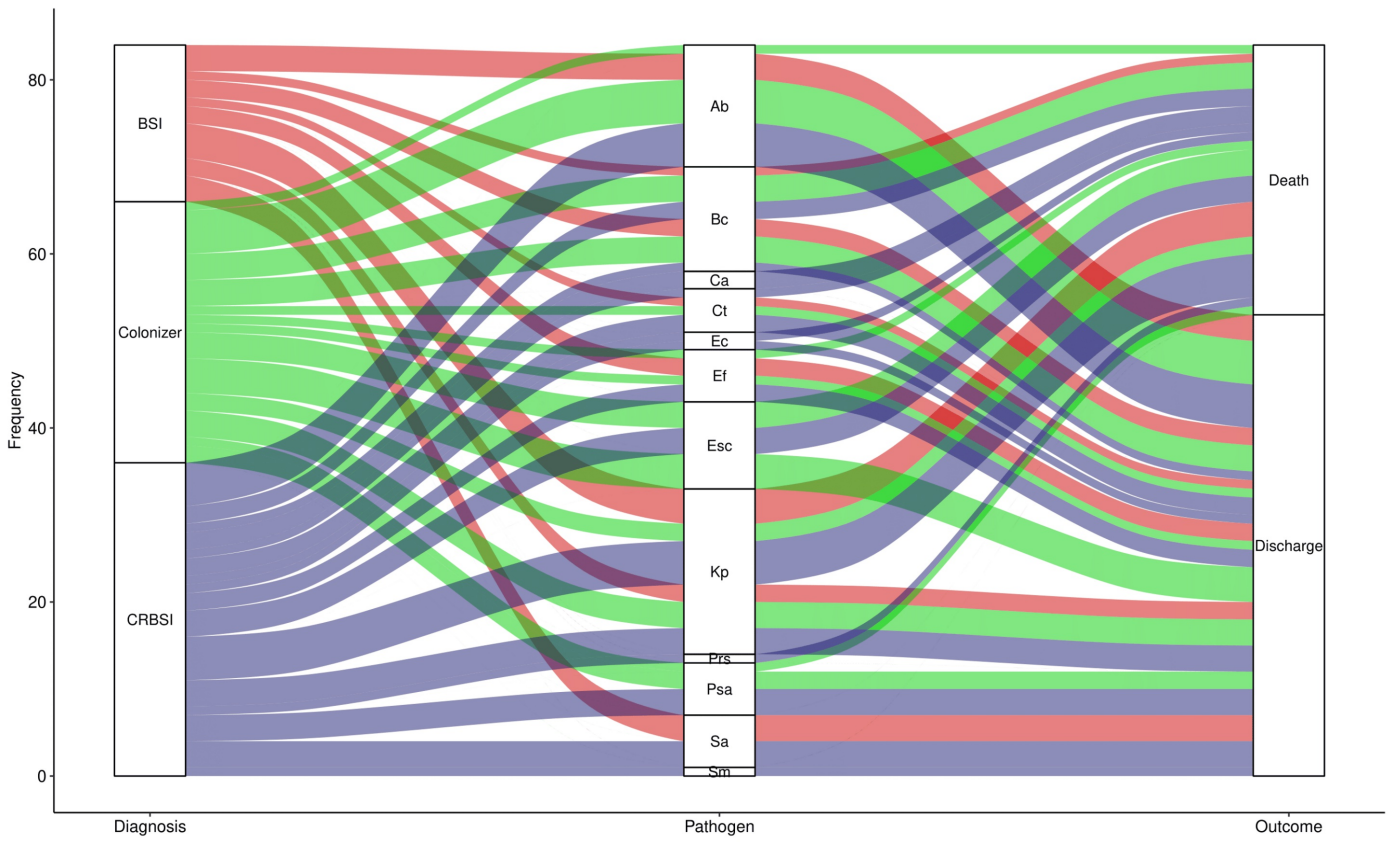
Pathogens involved and the fate of study participants The alluvial plot illustrates the association between the pathogen detected and the fate of the participants. The number of participants is shown on the y-axis. The diagnoses (i.e., CRBSI, colonizer, and BSI) are portrayed in different colours. The widths of bands denote the proportion of participants with the concerned parameters. CRBSI: catheter-related bloodstream infection; BSI: bloodstream infection; Ab: *Acinetobacter baumannii*; Bc: *Burkholderia cepacia*; Ca: *Candida albicans*; Ct: *Candida tropicalis*; Ec: *Enterobacter cloacae* complex; Ef: *Enterococcus faecium*; Esc: *Escherichia coli*; Kp: *Klebsiella pneumonia*; Prs: *Providencia stuartii*; Psa: *Pseudomonas aeruginosa*; Sa: *Staphylococcus aureus*; Sm: *Serratia marcescens*

The alluvial plot in Figure 2 showcases the pathogens involved and the fate of the study participants with CRBSI, colonizer, and BSI. All 12 species of pathogens encountered in the study population were associated with CRBSI. The most common pathogens causing death were *Burkholderia cepacia*, *Escherichia coli*, and *Klebsiella pneumoniae*. The fates of participants with different diagnoses were similar.

**Figure 3:**
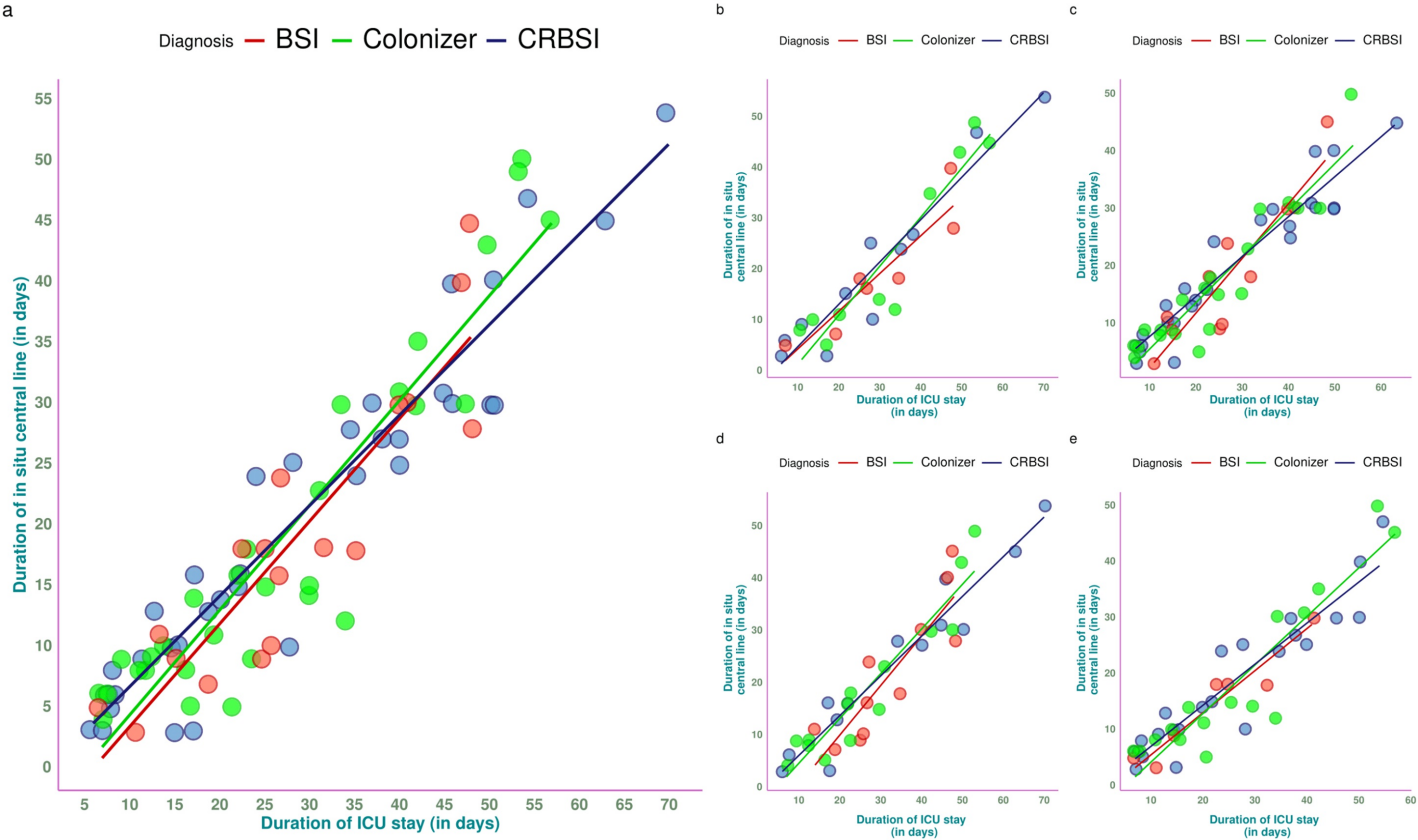
Correlation between durations of ICU stay and indwelling central venous catheter The correlation plots illustrate the association between the durations of ICU stay (shown on the x-axis) and the in situ central line (shown on the y-axis). The CRBSI, colonizer, and BSI participants are denoted in different colours. Figures 3a-3e show the correlation among the entire study population, females, males, the elderly, and young individuals, respectively. CRBSI: catheter-related bloodstream infections; BSI: bloodstream infections

The correlation plots in Figure 3 showcase the association between the durations of ICU stay and the in situ central line. There was a strong positive association (r = 0.63, 95% CI = 0.55-0.71, p < 0.001) between the durations of ICU stay and in situ central line of all participants (Figure 3a). The associations were also positive among those with BSI (r = 0.61, 95% CI = 0.52-0.71, p < 0.001), colonizer (r = 0.65, 95% CI = 0.53-0.78, p < 0.001), and CRBSI (r = 0.63, 95% CI = 0.51-0.74, p < 0.001). The association between these durations was also analyzed among females (Figure 3b), males (Figure 3c), elderly persons (Figure 3d), and young individuals (Figure 3e). The associations were similar irrespective of gender and age group.

Table 2 illustrates the antibiotic susceptibility pattern of three isolates of *Staphylococcus aureus* and two isolates of *Enterococcus faecium* among the participants with CRBSI. The three *Staphylococcus aureus* isolates were sensitive to vancomycin, linezolid, daptomycin, and tetracycline. These isolates were mainly resistant to erythromycin, clindamycin, and gentamicin. The two *Enterococcus faecium* isolates were sensitive to daptomycin, linezolid, tigecycline, and cotrimoxazole. These isolates were resistant to clindamycin, erythromycin, ciprofloxacin, levofloxacin, and tetracycline. Daptomycin and linezolid showed 100% sensitivity towards these Gram-positive cocci.

**Table 2:** Antibiotic susceptibility pattern of Gram-positive cocci among the participants with CRBSI Antibiotic susceptibility patterns (i.e., sensitive, intermediate, and resistant) of Gram-positive cocci are depicted as frequencies and proportions. CM: clindamycin; DAP: daptomycin; E: erythromycin; CIP: ciprofloxacin; GM: gentamicin; LVX: levofloxacin; LZD: linezolid; TEC: teicoplanin; TC: tetracycline; TGC: tigecycline; SXT: cotrimoxazole; VA: vancomycin; CRBSI: catheter-related bloodstream infections

Bacteria	CM	DAP	E	CIP	GM	LVX	LZD	TEC	TC	TGC	SXT	VA
*Staphylococcus aureus* (n = 3)												
Sensitive	1 (33%)	3 (100%)	0	1 (33%)	1 (33%)	2 (67%)	3 (100%)	2 (67%)	3 (100%)	2 (67%)	1 (33%)	3 (100%)
Intermediate	0	0	1 (33%)	1 (33%)	0	0	0	1 (33%)	0	1 (33%)	1 (33%)	0
Resistant	2 (67%)	0	2 (67%)	1 (33%)	2 (67%)	1 (33%)	0	0	0	0	1 (33%)	0
*Enterococcus faecium* (n = 2)												
Sensitive	0	2 (100%)	0	0	1 (50%)	0	2 (100%)	1 (50%)	0	2 (100%)	2 (100%)	1 (50%)
Intermediate	0	0	0	0	0	0	0	0	0	0	0	0
Resistant	2 (100%)	0	2 (100%)	2 (100%)	1 (50%)	2 (100%)	0	1 (50%)	2 (100%)	0	0	1 (50%)

Table 3 illustrates the antibiotic susceptibility pattern of five isolates of *Acinetobacter baumannii*, three isolates of *Burkholderia cepacia*, and three isolates of *Pseudomonas aeruginosa* among the participants with CRBSI. The *Acinetobacter baumannii* isolates were sensitive to ertapenem, colistin, tigecycline, meropenem, and imipenem. Colistin shows 100% sensitivity against the *Burkholderia cepacia* isolates. None of the antibiotics were completely sensitive or resistant to the *Pseudomonas aeruginosa* isolates. Ertapenem, tigecycline, and colistin showed the highest sensitivity towards these non-fermenters.

**Table 3:** Antibiotic susceptibility pattern of non-fermenters among the participants with CRBSI Antibiotic susceptibility patterns (i.e., sensitive, intermediate, and resistant) of non-fermenters are depicted as frequency and proportions. TZP: piperacillin-tazobactam; CRO: ceftriaxone; CFP: cefoperazone; FEP: cefepime; AN: amikacin; GM: gentamicin; MEM: meropenem; IPM: imipenem; ETP: ertapenem; SXT: cotrimoxazole; CAZ: ceftazidime; CXM: cefuroxime; CL: colistin; TGC: tigecycline; NA: not applicable; CRBSI: catheter-related bloodstream infections

Bacteria	TZP	CRO	CFP	FEP	AN	GM	MEM	IPM	ETP	SXT	CAZ	CXM	CL	TGC
*Acinetobacter baumannii* (n = 5)														
Sensitive	2 (40%)	2 (40%)	2 (40%)	2 (40%)	3 (60%)	3 (60%)	4 (80%)	4 (80%)	5 (100%)	2 (40%)	2 (40%)	2 (40%)	5 (100%)	5 (100%)
Intermediate	2 (40%)	1 (20%)	1 (20%)	2 (40%)	1 (20%)	1 (20%)	0	0	0	1 (20%)	1 (20%)	2 (40%)	0	0
Resistant	1 (20%)	2 (40%)	2 (40%)	1 (20%)	1 (20%)	1 (20%)	1 (20%)	1 (20%)	0	2 (40%)	2 (40%)	1 (20%)	0	0
*Burkholderia cepacia* (n = 3)														
Sensitive	1 (33%)	2 (67%)	1 (33%)	2 (67%)	0	1 (33%)	2 (67%)	2 (67%)	2 (67%)	1 (33%)	2 (67%)	1 (33%)	3 (100%)	2 (67%)
Intermediate	1 (33%)	1 (33%)	1 (33%)	1 (33%)	1 (33%)	1 (33%)	0	0	0	1 (33%)	0	0	0	0
Resistant	1 (33%)	0	1 (33%)	0	2 (67%)	1 (33%)	1 (33%)	1 (33%)	1 (33%)	1 (33%)	1 (33%)	2 (67%)	0	1 (33%)
*Pseudomonas aeruginos*a (n = 3)														
Sensitive	2 (67%)	NA	2 (67%)	2 (67%)	2 (67%)	2 (67%)	2 (67%)	2 (67%)	2 (67%)	NA	2 (67%)	1 (33%)	2 (67%)	2 (67%)
Intermediate	0		0	0	1 (33%)	1 (33%)	1 (33%)	1 (33%)	1 (33%)		0	1 (33%)	0	0
Resistant	1 (33%)		1 (33%)	1 (33%)	0	0	0	0	0		1 (33%)	1 (33%)	1 (33%)	1 (33%)

Table 4 illustrates the antibiotic susceptibility pattern of three isolates of *Escherichia coli*, eight isolates of *Klebsiella pneumoniae*, two isolates of *Enterobacter cloacae complex*, one isolate of *Providencia stuartii*, and one isolate of *Serratia marcescens* among the participants with CRBSI. The *Escherichia coli* isolates were mainly sensitive to tigecycline, piperacillin-tazobactam, amoxicillin-clavulanic acid, cefoperazone, meropenem, imipenem, amikacin, and gentamicin. These isolates showed resistance to cotrimoxazole and cefuroxime. The *Klebsiella pneumoniae* isolates were mainly sensitive to cefepime and tigecycline. These isolates were mostly resistant to ciprofloxacin, cefuroxime, piperacillin-tazobactam, ceftriaxone, meropenem, amoxicillin-clavulanic acid, cefoperazone, and imipenem. The *Enterobacter cloacae complex* isolates were mainly sensitive to piperacillin-tazobactam, gentamicin, and tigecycline. Their resistance was observed against cefuroxime, cefepime, imipenem, ciprofloxacin, and cotrimoxazole. The isolates of *Providencia stuartii* and *Serratia marcescens* were sensitive to cefepime, amikacin, cotrimoxazole, and tigecycline. Tigecycline and cefepime showed the highest sensitivity towards these Enterobacterales.

**Table 4:** Antibiotic susceptibility pattern of Enterobacterales among the participants with CRBSI Antibiotic susceptibility patterns (i.e., sensitive, intermediate, and resistant) of Enterobacterales are depicted as frequencies and proportions. AMC: amoxicillin-clavulanic acid; TZP: piperacillin-tazobactam; CXM: cefuroxime; CRO: ceftriaxone; CFP: cefoperazone; FEP: cefepime; MEM: meropenem; IPM: imipenem; AN: amikacin; GM: gentamicin; CIP: ciprofloxacin; SXT: cotrimoxazole; TGC: tigecycline; CRBSI: catheter-related bloodstream infections

Bacteria	AMC	TZP	CXM	CRO	CFP	FEP	MEM	IPM	AN	GM	CIP	SXT	TGC
*Escherichia coli *(n = 3)													
Sensitive	2 (67%)	2 (67%)	0	0	2 (67%)	1 (33%)	2 (67%)	2 (67%)	2 (67%)	2 (67%)	0	0	3 (100%)
Intermediate	0	1 (33%)	1 (33%)	1 (33%)	1 (33%)	1 (33%)	0	0	1 (33%)	0	2 (67%)	1 (33%)	0
Resistant	1 (33%)	0	2 (67%)	2 (67%)	0	1 (33%)	1 (33%)	1 (33%)	0	1 (33%)	1 (33%)	2 (67%)	0
*Klebsiella pneumoniae* (n = 8)													
Sensitive	1 (13%)	0	0	0	1 (13%)	5 (62%)	2 (25%)	1 (13%)	1 (13%)	3 (37%)	0	1 (13%)	5 (62%)
Intermediate	2 (25%)	2 (25%)	2 (25%)	2 (25%)	2 (25%)	1 (13%)	0	2 (25%)	3 (37%)	1 (13%)	1 (13%)	3 (37%)	1 (13%)
Resistant	5 (62%)	6 (75%)	6 (75%)	6 (75%)	5 (62%)	2 (25%)	6 (75%)	5 (62%)	4 (50%)	4 (50%)	7 (87%)	4 (50%)	2 (25%)
*Enterobacter cloacae *complex(n = 2)													
Sensitive	1 (50%)	2 (100%)	0	1 (50%)	1 (50%)	1 (50%)	1 (50%)	1 (50%)	1 (50%)	2 (100%)	1 (50%)	0	2 (100%)
Intermediate	1 (50%)	0	1 (50%)	1 (50%)	1 (50%)	0	1 (50%)	0	1 (50%)	0	0	1 (50%)	0
Resistant	0	0	1 (50%)	0	0	1 (50%)	0	1 (50%)	0	0	1 (50%)	1 (50%)	0
*Providencia stuartii *(n = 1)													
Sensitive	0	0	0	0	0	1 (100%)	1 (100%)	0	1 (100%)	0	0	1 (100%)	1 (100%)
Intermediate	1 (100%)	0	1 (100%)	0	1 (100%)	0	0	1 (100%)	0	0	1 (100%)	0	0
Resistant	0	1 (100%)	0	1 (100%)	0	0	0	0	0	1 (100%)	0	0	0
*Serratia marcescens* (n = 1)													
Sensitive	0	0	0	0	1 (100%)	1 (100%)	0	0	1 (100%)	0	0	1 (100%)	1 (100%)
Intermediate	0	1 (100%)	0	1 (100%)	0	0	1 (100%)	1 (100%)	0	1 (100%)	1 (100%)	0	0
Resistant	1 (100%)	0	1 (100%)	0	0	0	0	0	0	0	0	0	0

Table 5 illustrates the antibiotic susceptibility pattern of three *Candida tropicalis* and two isolates of *Candida albicans* among the participants with CRBSI. The *Candida tropicalis* isolates were mainly sensitive to caspofungin, micafungin, flucytosine, and voriconazole. These isolates were resistant to fluconazole. The *Candida albicans* isolates were 100% sensitive to caspofungin, flucytosine, micafungin, and voriconazole. Caspofungin and micafungin showed 100% sensitivity towards these five isolates.

**Table 5:** Antibiotic susceptibility pattern of yeast among the participants with CRBSI Yeast's antibiotic susceptibility patterns (i.e., sensitive, intermediate, and resistant) are depicted as frequencies and proportions. CRBSI: catheter-related bloodstream infections

Yeast	Caspofungin	Fluconazole	Flucytosine	Micafungin	Voriconazole	Amphotericin B
*Candida tropicalis* (n = 3)						
Sensitive	3 (100%)	0	2 (67%)	3 (100%)	2 (67%)	1 (33%)
Intermediate	0	1 (33%)	0	0	1 (33%)	1 (33%)
Resistant	0	2 (67%)	1 (33%)	0	0	1 (33%)
*Candida albicans *(n = 2)						
Sensitive	2 (100%)	1 (50%)	2 (100%)	2 (100%)	2 (100%)	1 (50%)
Intermediate	0	1 (50%)	0	0	0	1 (50%)
Resistant	0	0	0	0	0	0

## Discussion

In this cross-sectional study, 84 (7.1%) of 1183 patients admitted to the medicine ICU were suspected to have CRBSI. The study population's median age was 59.5 (49.5-67.3) years. Twenty-eight (33.3%) participants were females. Of the 84 participants, 36 (42.8%) had CRBSI and 18 (21.6%) had BSI. The remaining 30 (35.6%) subjects were colonizers. Enterobacterales (e.g., *Klebsiella pneumoniae* and *Escherichia coli*), non-fermenters (e.g., *Acinetobacter baumannii*, *Burkholderia cepacia*, and *Pseudomonas aeruginosa*), Gram-positive cocci (e.g., *Staphylococcus aureus*), and yeast (e.g., *Candida tropicalis* and *Candida albicans*) were the most common microorganisms found in the culture reports. Our findings concorded with previous studies [16-18].

Most of our study participants were young (aged < 60 years) and male. The internal jugular vein (34, 40.5%) was the most common cannulation site, followed by the femoral vein (28, 33.3%) and the subclavian vein (22, 26.2%). Our observations matched the studies by Hafeez et al. [5] and Costantine et al. [16]. After ICU admission, most of the subjects were cannulated for volume resuscitation and total parenteral nutrition. One-fourth of the population developed septic shock during their ICU stay. All these factors might have prolonged the ICU stay and the indwelling catheter. Immunocompromised state, lengthy hospitalization, and polypharmacy facilitate CRBSI and worsen the prognosis [18,22,23]. The incidences of CRBSI in our study subjects were similar for all three insertion sites. Recent studies advocate that the variation in insertion sites is not linked to the level of risk of CRBSI [24,25].

Colonization of microorganisms in the catheter and their systemic dissemination might involve one or more of the following routes: invasion of the skin insertion site, contamination of infused fluid through the catheter, hematogenous spread, and contamination of the hub [18,26]. *Klebsiella pneumoniae* (8, 22.2%) and *Acinetobacter baumannii* (5, 13.9%) were the most frequent pathogens responsible for CRBSI in 36 participants. The other common pathogens were *Burkholderia cepacia* (3, 8.3%), *Candida tropicalis* (3, 8.3%), *Escherichia coli* (3, 8.3%), *Pseudomonas aeruginosa* (3, 8.3%), and *Staphylococcus aureus* (3, 8.3%). The tigecycline sensitivity of *Escherichia coli* and *Klebsiella pneumoniae* specimens was high. *Pseudomonas aeruginosa*, *Burkholderia cepacia*, and *Acinetobacter baumannii* specimens were primarily susceptible to tigecycline, ertapenem, and colistin. Daptomycin, vancomycin, and linezolid showed 100% sensitivity in *Staphylococcus aureus* isolates. Similarly, *Candida tropicalis* isolates exhibited 100% sensitivity to echinocandins (e.g., micafungin and caspofungin).

The major strengths of our study were data presentation through the mosaic and alluvial plots, as well as an illustration of the AST patterns. This study also had a few limitations. First, the sample size of CRBSI was less to assess the relation between the insertion site and the pathogen. Second, we did not investigate the association between the antibiotics used in the ICU and the duration of an indwelling catheter. Third, we could not analyze the relationship between the AST findings and outcome. Fourth, we did not evaluate any risk factors for the development of CRBSI in ICU patients.

## Conclusions

The major pathogens causing CRBSI in our research participants were *Klebsiella pneumoniae*, *Acinetobacter baumannii*, *Burkholderia cepacia*, *Pseudomonas aeruginosa*, *Escherichia coli*, *Staphylococcus aureus*, and *Candida tropicalis*. We also found specimens of *Providencia stuartii* and *Serratia marcescens* in the patients with CRBSI. Tigecycline has a high susceptibility to Gram-negative bacteria. Specimens of *Staphylococcus aureus* showed susceptibility to daptomycin, vancomycin, and linezolid. Echinocandin sensitivity was observed in the *Candida tropicalis* isolates. The duration of ICU stay and indwelling catheters are positively associated. We warrant more prospective studies with a larger sample size to investigate the microorganisms causing CRBSI and their AST patterns.
